# A Neural Sensor with a Nanocomposite Interface for the Study of Spike Characteristics of Hippocampal Neurons under Learning Training

**DOI:** 10.3390/bios12070546

**Published:** 2022-07-21

**Authors:** Shihong Xu, Yu Deng, Jinping Luo, Yaoyao Liu, Enhui He, Yan Yang, Kui Zhang, Longze Sha, Yuchun Dai, Tao Ming, Yilin Song, Luyi Jing, Chengyu Zhuang, Qi Xu, Xinxia Cai

**Affiliations:** 1 State Key Laboratory of Transducer Technology, Aerospace Information Research Institute, Chinese Academy of Sciences, Beijing 100190, China; xushihong18@mails.ucas.ac.cn (S.X.); jpluo@mail.ie.ac.cn (J.L.); liuyaoyao20@mails.ucas.ac.cn (Y.L.); heenhui_iecas@163.com (E.H.); yangyan201@mails.ucas.ac.cn (Y.Y.); 15595670837@163.com (K.Z.); hongri1991@126.com (Y.D.); mingtao17@mails.ucas.ac.cn (T.M.); ylsong@mail.ie.ac.cn (Y.S.); jingluyi20@mails.ucas.ac.cn (L.J.); 2 School of Electronic, Electrical and Communication Engineering, University of Chinese Academy of Sciences, Beijing 100049, China; 3 State Key Laboratory of Medical Molecular Biology, Institute of Basic Medical Sciences, Chinese Academy of Medical Sciences and Peking Union Medical College, Beijing 100005, China; dengyuandf@outlook.com (Y.D.); shalz_pumc@163.com (L.S.); qixu@vip.sina.com (Q.X.); 4 Department of Orthopaedics, Ruijin Hospital, Shanghai Jiao Tong University School of Medicine, Shanghai 200025, China; zhuangchengyu@msn.com

**Keywords:** neural sensor, porous graphene, PEDOT, neurons, learning

## Abstract

Both the cellular- and population-level properties of involved neurons are essential for unveiling the learning and memory functions of the brain. To give equal attention to these two aspects, neural sensors based on microelectrode arrays (MEAs) have been in the limelight due to their noninvasive detection and regulation capabilities. Here, we fabricated a neural sensor using carboxylated graphene/3,4-ethylenedioxythiophene:polystyrenesulfonate (cGO/PEDOT:PSS), which is effective in sensing and monitoring neuronal electrophysiological activity in vitro for a long time. The cGO/PEDOT:PSS-modified microelectrodes exhibited a lower electrochemical impedance (7.26 ± 0.29 kΩ), higher charge storage capacity (7.53 ± 0.34 mC/cm^2^), and improved charge injection (3.11 ± 0.25 mC/cm^2^). In addition, their performance was maintained after 2 to 4 weeks of long-term cell culture and 50,000 stimulation pulses. During neural network training, the sensors were able to induce learning function in hippocampal neurons through precise electrical stimulation and simultaneously detect changes in neural activity at multiple levels. At the cellular level, not only were three kinds of transient responses to electrical stimulation sensed, but electrical stimulation was also found to affect inhibitory neurons more than excitatory neurons. As for the population level, changes in connectivity and firing synchrony were identified. The cGO/PEDOT:PSS-based neural sensor offers an excellent tool in brain function development and neurological disease treatment.

## 1. Introduction

The tremendous number of connections between neurons through synapses is responsible for various brain functions, such as sensory perception [1,2], motor action [3], and learning and memory [4,5].

Among them, the learning function related to synaptic plasticity has attracted much attention with the rise of artificial intelligence [6]. While some researchers have conducted in-depth studies on the electrophysiological characteristics of neural network learning in vitro [7,8], better tools are still needed in order to further understand the neural dynamics at the cell and network level, thereby uncovering the mechanism of brain learning function.

As a neural sensor, microelectrode arrays (MEAs) can sense changes in the electrical activity of neurons, and these changes detected by MEA have played an important role in drug screening [9,10], toxicity research [11], disease diagnosis [12], and mechanism research [13,14,15]. In recent years, neural sensors have gradually gained recognition in the study of brain learning functions. Li et al. [16] used a neural sensor to verify the significance of synchronized bursts of neurons in the learning process. Feber et al. [7] also found that electrical stimulation caused the network connections on the neural sensors to form a new balance after learning.

For in vitro neurons, electrical stimulation is a routine technique that induces neuronal plasticity [17]. The ideal neural sensor for brain mechanism research should simultaneously possess extraordinary recording and stimulation properties. Furthermore, the neural interface for the sensor should have long-term stability and should be non-toxic to organisms. Precious metals such as Pt or Ir [18] have been widely used as interface materials for microelectrodes in research on implantable MEA and ex vivo MEA. However, the disadvantages of metal interfaces are their limited abilities to safely stimulate nerves [19], the high inherent noise level of nerve recordings [20], and the mechanical mismatch between the electrodes and surrounding cells [21].

In the past ten years, conductive polymers including polypyrrole (PPy) [22] and poly(3,4-ethylenedioxythiophene) (PEDOT) have attracted much attention as interface materials. In particular, PEDOT has been widely used in neural interfaces due to its biocompatibility and chemical stability [23,24,25,26]. The electrochemical deposition of PEDOT forms a positively charged framework, which provides accommodation for negatively charged dopants to reach charge balance. Additionally, polystyrenesulfonate (PSS) is the most common dopant for PEDOT. Studies have shown that when PEDOT is involved in a long-term oxidation reduction reaction, it is liable to deform and crack. As an effective solution, a composite material of PEDOT and mechanically strong carbonaceous materials can greatly increase stability [27]. Graphene has attracted much attention in the field of neurological applications due to its high electrical conductivity and mechanical stability [28,29]. By chemical functionalization with hydroxyl or carboxyl groups, it is easy to endow graphene with new properties in order to further study and expand its application fields [30]. Therefore, we believe that PEDOT:PSS and carboxylated graphene (cGO) are promising candidates for the development of outstanding neural interfaces.

Nanocomposites composed of graphene and PEDOT have been widely used as neural interfaces in the field of nerves and their intersections. Hsiao et al. [31] utilized graphene and PEDOT to construct a bioelectronic interface that can be used to manipulate the attachment and orientation of human mesenchymal stem cells. He et al. [32] used a nanocomposite composed of graphene and PEDOT to improve the sensitivity of MEA in detecting dopamine, and they used MEA to detect the quantized release of dopamine from dopaminergic neurons derived from embryonic stem cells. This work combines graphene and PEDOT to develop an innovative MEA with a high spatiotemporal resolution detection (low impedance, small phase delay) and excellent electrical control capability, which also has good biocompatibility and long-term stability.

Here, MEAs were coated with three different materials, i.e., Pt, CNT/PEDOT:PSS, and cGO/PEDOT:PSS were coated onto MEAs, and hippocampal neurons were cultured on MEAs to fabricate a sensor for studying the learning functions of neural network. The performance of the sensor was evaluated in vitro using electrochemical impedance spectroscopy (EIS), scanning electron microscope (SEM), cyclic voltammetry (CV), and voltage transient test. Moreover, the neural sensor successfully altered the spiking activity of hippocampal neurons via electrical stimulation. At the cellular level, we found that the firing frequency of both excitatory and inhibitory neurons increased after electrical stimulation, but the increase in the firing frequency of inhibitory neurons was more pronounced. At the network level, the electrical stimulation changed the speed of information transmission between the two groups of neurons.

## 2. Materials and Methods

### 2.1. Reagents and Apparatus

Polystyrenesulfonate was purchased from HEROCHEM (Shanghai, China). 3,4-ethylenedioxythiophene (EDOT) was purchased from Aladdin (Shanghai, China). Carboxylated graphene dispersion was purchased from XFNANO (Nanjing, China). Carboxylated carbon nanotube (CNT) powder was purchased from XFNANO (China). Phosphate-buffered saline (PBS, 0.1 M, PH 7.4) and glutamate were purchased from sigma (Shanghai, China). HBSS buffer, DNase, papain, DMEM buffer and Neurobasal Plus Medium were obtained from Sigma-Aldrich (St. Louis, MO, USA), and cytarabine was obtained from Thermo Fisher (Waltham, MA, USA).

The electrodeposition of materials and the characterization of microelectrode properties were carried out in an electrochemical workstation (Gamry Reference 600, Gamry Instruments, Warminster, PA, USA). The electrophysiological signals were recorded using a 128-channel neuron data recording system (Blackrock Microsystems, Salt Lake City UT, USA). Other apparatuses included an ultrasonic cleaner (KH200KDB, Hechuang, Suzhou, China), preamplifier (Blackrock Microsystems, USA), CO_2_ incubator (Themo Fisher, Waltham, MA, USA), dual-channel electrophysiological electrical stimulator (MultiChannel, Baden-Württemberg, Reutlingen, Germany), and oscilloscope (TPS2024, Tektronix, Beaverton, OR, USA).

### 2.2. Fabrication of Neural Sensor and Cell Culture

In the preparation of neural sensors, the two aspects of device fabrication and cell culture need to be considered.

In this work, we fabricated a MEA with 59 microelectrodes and 1 counter electrode. The diameter of the microelectrodes was 30 μm, the distance between adjacent microelectrodes was 200 μm, and the microelectrode arrangement of the MEA was as shown in Figure S1A.

**Figure 1:**
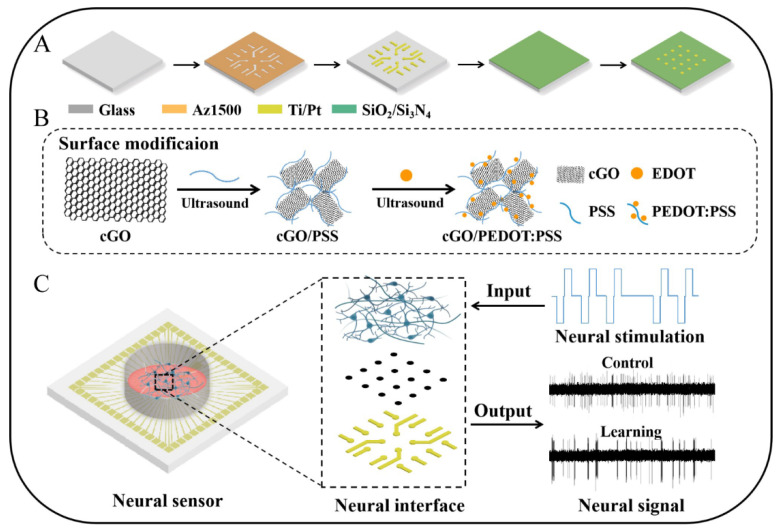
Fabrication and mechanism of the neural sensor. (**A**) Manufacturing process of the neural sensor. (**B**) Modification of the neural interface with nanocomposites. (**C**) Working principle of the sensor. Neurons can grow on the neural sensor, where the interface is the core component. Through the vital link, the integrated system can process the input stimulation information and output corresponding neural signals.

The MEA fabrication process is shown in Figure 1A. Before the process, the substrate was rinsed with piranha liquid for surface cleaning. Then, AZ1500 photoresist was spin-coated onto the surface of the substrate at a thickness of about 1.5 μm. The pattern of sites, wires and contact pads was transferred to the substrate by photolithography and development. Subsequently, a Cr seed layer with a thickness of 30 nm was sputtered on the surface of the substrate, followed by a 250 nm Au thin film layer. The excess Cr/Au thin film layer was removed by a lift-off process, and the required conductive layers such as sites and wires were left on the surface of substrate. Next, the plasma-enhanced chemical vapor deposition (PECVD) method was used to cover the surface of the substrate with an insulation layer of SiO_2_/Si_3_N_4_ (300 nm/500 nm). In the PECVD process, the temperature was 130 °C, the pressure was 1.03 Pa, and the power was 350 W. Finally, a mask was formed by a second photolithography process, and the sites and contact pads on the MEA were exposed by SF_6_ gas etching for about 15 min. After preparing the MEA, we used silica gel to combine the MEA with a ring for cell culture.

To isolate hippocampal neurons, fetuses of pregnant cancer institute mice were dissected under a microscope and placed in pre-chilled HBSS buffer. Subsequently, the fetal hippocampus was isolated and dissociated in DMEM buffer for 0.5 h. After gently blowing away the tissue with a 1000 μL micropipette, the cell-containing solution was transferred into a sterile 10 mL centrifuge tube. Next, the dissociated cells were resuspended in medium after being centrifuged at 100 g for 200–300 s. Finally, the resuspended cells were counted with a hemocytometer, and the cells were cultured at a density of 5 × 10^5^ cells/mL onto the neural sensor. Before performing the experiments, the medium was changed every 3–4 days. During this period, neurons were kept in a constant-temperature (37 °C) cell incubator with 95% humidity at 5% CO_2_. All the protocols complied with the regulations of Institutional Animal Care and Use Committee at Aerospace Information Research Institute, Chinese Academy of Science (AIRCAS).

### 2.3. Modification of Neural Interface

The neural interface is the bridge that connects electronic devices and biological tissues. An excellent neural interface can greatly improve the detection and regulation performance of a neural sensor. In this study, we used the cGO/PEDOT:PSS, a nanocomposite material, to perfect the neural interface.

The cGO/PEDOT:PSS was electrochemically deposited on the MEA sites (Figure 1B). First, 0.1 M of PSS was added to 8 mL of cGO (2 mg/mL) dispersion and sonicated for 0.5 h to obtain a cGO/PSS mixture. Then, 0.02 M EDOT was added to the mixed solution, and it was sonicated again for 0.5 h to acquire the final nanocomposite mixed solution. Finally, the MEA was installed into our designed interface circuit (Figure S1B), and the interface circuit was connected to the electrochemical workstation. The cGO/PEDOT:PSS was precisely deposited onto the surfaces of all microelectrodes by CV for a total of 15 cycles, where the potential range was 0 to 0.95 V and the scan rate was 0.1 V/s. A Pt counter electrode and Ag/AgCl reference electrode were adopted.

The electrochemical deposition steps of CNT/PEDOT:PSS were similar to cGO/PEDOT:PSS. First, 1 g PSS and 16 mg CNT powder were added to 8 mL of deionized water, and then, the mixture was sonicated for 0.5 h to obtain a CNT/PSS mixture. Next, 1 mg EDOT was added to the mixed solution, and it was sonicated again for 0.5 h to obtain a mixed solution of CNT/PEDOT:PSS. Finally, CNT/PEDOT:PSS was deposited onto the electrode by CV, and the parameters of the CV were consistent with those used for cGO/PEDOT:PSS.

### 2.4. Protocol for Learning Training Hippocampal Neurons

A bipolar voltage pulse sequence starting in negative phase is an effective stimulation method for encouraging neurons to learn [16]. In hippocampal neuron learning training experiments, we used a bipolar pulse sequence with a frequency of 1 Hz, an amplitude of ±300 mV, and a pulse width of 200 μs. We successively applied 6 electrical stimulation sequences to hippocampal neurons cultured in vitro, where each sequence lasted 5 min, and the interval between electrical stimulation sequences was 20 min (Figure 1C and Figure S2). At the same time, we recorded electrophysiological signals throughout the process.

### 2.5. Data Processing and Analysis

To monitor electrical activity in a stable state, the neural sensor with cultured neurons was placed in the testing environment for 30 min before recording. Additionally, we placed the MEA on a 37 °C hot plate to keep the temperature constant after leaving the cell incubator. For all recording sites, the electrical signals of the neurons were amplified and sampled in real time at a sampling rate of 30 kHz. A band-pass filter (250 Hz to 5 kHz) was used to obtain the action potential of neurons, and a low-pass filter (250 Hz) was used to obtain the local field potential (LFP). The Cerebus Central Software (Blackrock Microsystems, Utah, USA) was used to record the experimental data.

In the microelectrode characterization experiment, we used 3 MEAs produced in the same batch for the characterization of Pt, PEDOT:PSS/CNT, and PEDOT:PSS/cGO. The performance parameters of each microelectrode were calculated from the average results of 5 microelectrodes. The recorded neuronal data were obtained from microelectrodes on the same MEA.

Data were calculated as mean ± standard error of mean. The mean values were compared using a two-tailed T test for two groups. A statistical significance of *p* < 0.05 was set for all analyses.

## 3. Results and Discussion

### 3.1. Morphology and Recording Characteristics of Neural Sensor

**Figure 2:**
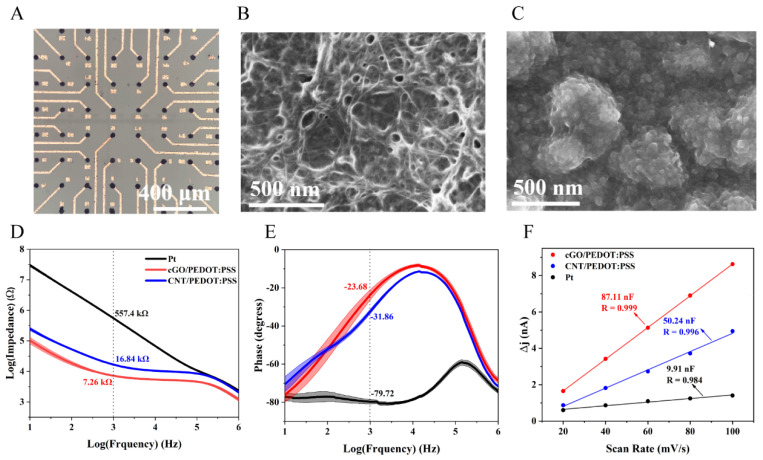
The morphological and electrical properties of the neural sensor. (**A**) Optical microscope image of modified MEA. (**B**) SEM image of the microelectrode with CNT/PEDOT:PSS material modification. (**C**) SEM image of the microelectrode with cGO/PEDOT:PSS material modification. (**D**) Impedance of the three neural interfaces in the range of 10 Hz to 1 MHz. (**E**) Phase properties of these three neural interfaces at 10 Hz to 1 MHz. (**F**) The double layer capacitance of the neural interfaces.

Observing the microelectrode sites of the neural sensor under SEM, we found that before the electrodeposition, the interface surface was smooth and appeared dark gray (Figure S3A). After the electrodeposition of CNT/PEDOT:PSS or cGO/PEDOT:PSS, the neural interface became rough, and obvious pore-like structures could be observed (Figure 2B,C). Further observation revealed that the neural interface of CNT/PEDOT:PSS showed a net-like nanostructure (Figure 2B and Figure S3B), while cGO/PEDOT:PSS showed a sheet-like structure (Figure 2C and Figure S3C). Graphene and carbon nanotubes were embedded in PEDOT:PSS through chemical coupling, forming a complex 3D nano porous structure. Studies have shown that the recording performance of neural sensors is closely related to the roughness of the interface [33].

To evaluate the performance of the interface materials, it is often necessary to analyze the surface area that participates in electrochemical reactions. Therefore, researchers put forward the concept of an electrochemical active surface area (ECSA). At present, the double capacitive layer measurement method (C_dl_), which is recognized as a reasonable method, can be used for testing ECSA. The general rule is that an electrode with a large surface area will be beneficial for capacitive energy storage. We used this method to roughly estimate the ECSA of the three neural interfaces of Pt, CNT/PEDOT:PSS and cGO/PEDOT:PSS. In the absence of faradaic current, the C_dl_ was measured in the CV experiments in a solution containing only a supporting electrolyte and used for the estimation of the active area of the interface (Figure S4). The cyclic voltammetry curves were between 0 and 0.6 V at different scan rates from 20 to 100 mV/s. The C_dl_ was determined by measuring the capacitive current associated with double-layer charging from the scan rate dependence of the cyclic voltammogram. The results showed that the C_dl_ of Pt was only 9.91 nF, while that of CNT/PEDOT:PSS and cGO/PEDOT:PSS increased to 50.24 and 87.11 nF, respectively (Figure 2F). This showed that cGO/PEDOT:PSS had the largest ECSA.

Next, the EIS of these three neural interfaces was compared. As shown in Figure 2D,E, in the range of 10 Hz to 1 MHz, the impedance and phase delay of Pt, CNT/PEDOT:PSS and cGO/PEDOT:PSS were compared through a bode diagram. The results showed that in the full frequency domain, the two nanocomposites of CNT/PEDOT:PSS and cGO/PEDOT:PSS had greatly reduced impedance and phase delay. Additionally, this could also be verified from the size of the semicircle in the nyquist impedance spectrum (Figure S5B). Since the center frequency of neuron activity is 1 kHz [34], we calculated the impedance and phase delay of the three interfaces at this frequency. Their impedances were 557.4 ± 22.37 kΩ (Pt), 16.84 ± 0.70 kΩ (CNT/PEDOT:PSS), and 7.26 ± 0.29 kΩ (cGO/PEDOT: PSS), and the phases were −79.72 ± 1.20° (Pt), −31.86 ± 0.98° (CNT/PEDOT:PSS), and −23.68 ± 2.68° (cGO/PEDOT:PSS) (*n* = 5). cGO/PEDOT:PSS showed the lowest impedance and the smallest phase delay.

In addition, good biocompatibility is an important feature for long-term detection. In experiments with hippocampal cultures, we found that neurons could attach to the microelectrode stably and densely for 2–4 weeks (Figure S6A), which indicated the good biocompatibility of cGO/PEDOT:PSS.

In summary, the cGO/PEDOT:PSS neural interface had the best comprehensive detection performance, including a low impedance, small phase delay, high S/N, and good biocompatibility, which are crucial for the acquisition of weak neural information with the sensor.

### 3.2. Stimulation Characteristics of Neural Sensor

**Figure 3:**
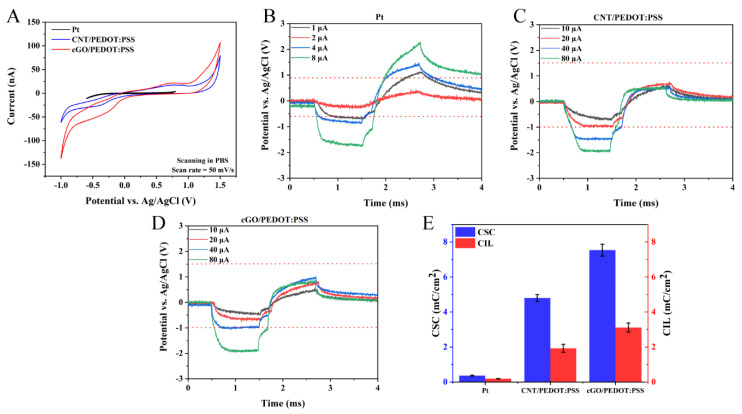
The stimulation performance of the neural sensor. (**A**) The CSCs of Pt-, CNT/PEDOT:PSS-, and cGO/PEDOT:PSS-coated electrodes were measured by CV. (**B**) Measurement of CIL of Pt electrodes via the voltage transient method. (**C**) Measurement of CIL of CNT/PEDOT:PSS-modified electrodes. (**D**) Measurement of CIL of cGO/PEDOT:PSS-modified electrodes. (**E**) CSC and CIL of Pt, CNT/PEDOT:PSS and cGO/PEDOT:PSS (*n* = 5).

The charge storage capacity (CSC) of the neural interface is the chief indicator for evaluating the stimulation performance of a sensor. CSC can be obtained from the integral calculation of the curve through slow potential scanning in the safe potential window. Generally, the safety potential window of metal electrodes is from −0.6 to 0.8 V (Figure S7A), but electrodes with a carbon material coating usually show a larger safety potential window than metal electrodes [20,35]. As shown in Figure S7B, the safety potential window of the cGO/PEDOT:PSS electrodes extended from −1 to 1.5 V. Within the safety window, we calculated and compared the CSC of the interface for the three materials. The CSC of the interface of Pt, CNT/PEDOT:PSS, and cGO/PEDOT:PSS calculated from the CV measurements were 0.37 ± 0.03, 4.80 ± 0.20 and 7.53 ± 0.34 mC/cm^2^, respectively (*n* = 5) (Figure 3A,E).

CSC generally reflects the stimulation performance under the slow response of the interface, whereas the transient stimulation performance of the interface is also important in neural applications. The charge injection limit (CIL) is defined as the instantaneous maximum amount of charge that can be injected without exceeding the safety potential window, which limits the maximum safe current stimulation. As shown in Figure 3B–D, the CILs of Pt, CNT/PEDOT:PSS and cGO/PEDOT:PSS were measured with an oscilloscope using the method of gradually increasing the pulsed current (Figure S8). The red dashed line in the figure represents the safety potential window, beyond which the polarized response to the cell will be damaged. The CILs of the neural interfaces of Pt, CNT/PEDOT:PSS and cGO/PEDOT:PSS were 0.19 ± 0.01, 1.93 ± 0.23, and 3.11 ± 0.25 mC/cm^2^, respectively (*n* = 5) (Figure 3B–E). These results indicate that cGO/PEDOT:PSS exhibited the best charge transfer ability. As shown in Table S1, the recording and stimulation performance of the cGO/PEDOT:PSS-modified microelectrodes far outperformed those of the traditional metal microelectrodes.

### 3.3. Stability of Neural Interface

As for studying and developing brain functions in vitro on neural sensors, the long-term stability plays a vital role in the accuracy of the research results. However, the recording and stimulation performances of the neural sensors will inevitably decline in long-term experiments. This can mainly be attributed to two aspects. One is the physical change that takes place at the surface of the microelectrode, such as coating delamination, corrosion, and changes in specific surface area caused by falling coating materials [36]. The other is the biological effect produced by the cells during culture.

**Figure 4:**
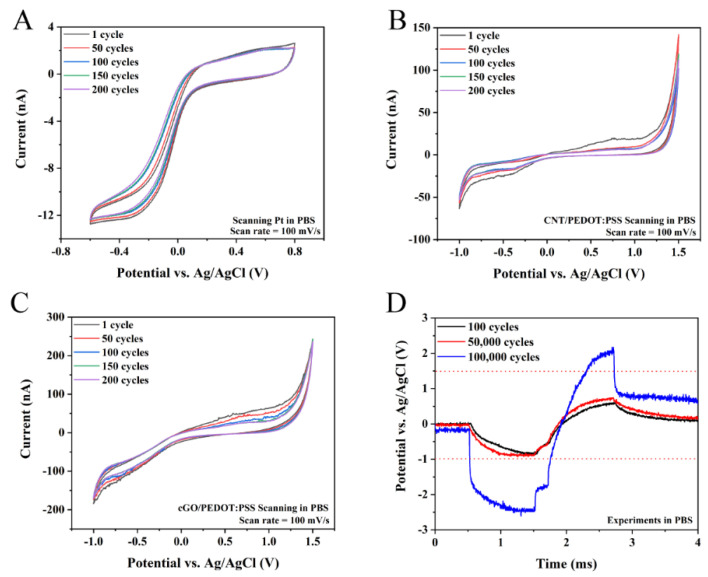
Stability of the interface. (**A**) The CV scanning curve of the Pt-based interface after the 1st, 50th, 100th, and 200th scan cycles. (**B**) The CV scanning curve of the CNT/PEDOT:PSS-modified interface after the 1st, 50th, 100th, and 200th scan cycles. (**C**) The CV scanning curve of the cGO/PEDOT:PSS-modified interface after the 1st, 50th, 100th, and 200th scan cycles. (**D**) The stability of the interface (cGO/PEDOT:PSS) under long-term stimulation.

A total of 200 CV scans (scan rate = 100 mV/s) were performed on the three interfaces of Pt, CNT/PEDOT:PSS and cGO/PEDOT:PSS to evaluate their electrical stability under a slow response. Pt was deposited on the MEA by sputtering, and the CSC of Pt did not change during the scanning process (Figure 4A). However, the CSC of both CNT/PEDOT:PSS and cGO/PEDOT:PSS showed some loss in performance during the first 100 CV scans (Figure 4B,C). After 100 CV scans, the interfacial CSC of the two materials tended to stabilize. This was because the electrochemically deposited PEDOT was not dense enough, and part of the PEDOT cracked in the early stage of the CV (Figure S9), resulting in a decrease in CSC. After applying 100, 50,000, and 100,000 cycles of cathode-first bipolar current pulses, we tested the voltage transients of cGO/PEDOT:PSS (Figure 4D). The results showed that the electrical stimulation properties of the neural interfaces were maintained at low pulse counts (below 50,000 cycles). The transient electrical stimulation performance of cGO/PEDOT:PSS started to degrade when pulses were applied over 100,000 cycles.

Next, we analyzed the recording performance of the neural sensor before and after 21 days of culture. First, the morphologies of the nerve interface before and after the culture were compared, and it was found that after 21 days of culture, only cellular metabolites were left on the microelectrode surface, but the structure of the coating did not change visibly (Figure S6A). Then, the impedance of the cGO/PEDOT:PSS-modified microelectrodes before and after the culture was measured. Although the impedance increased from 7.26 ± 0.29 kΩ (before culture) to 11.82 ± 1.50 kΩ (after culture) (*n* = 5) (Figure S6B), the cGO/PEDOT:PSS-modified microelectrodes were still able to record the electrical activity of the neurons with a high quality.

### 3.4. Response of Hippocampal Neurons to Electrical Stimulation

**Figure 5:**
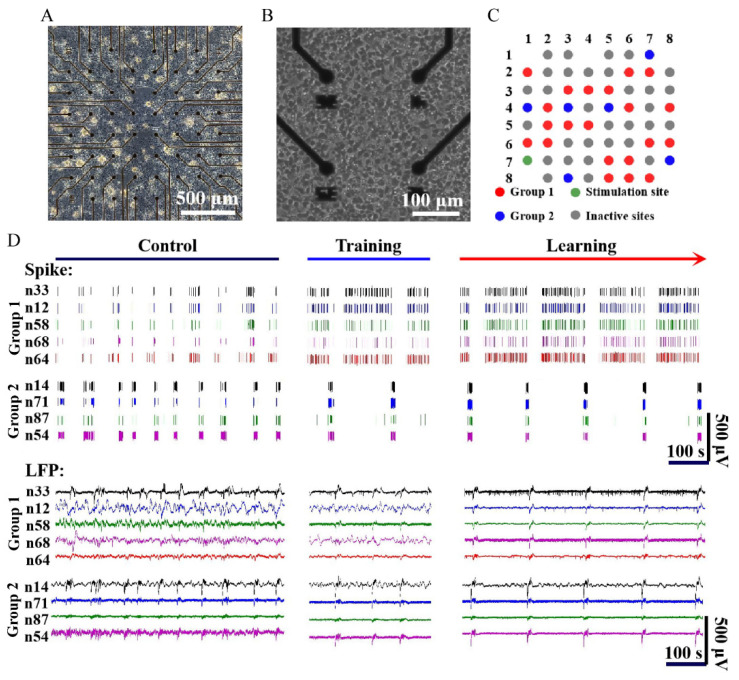
Electrophysiology signals from the dissociated hippocampal culture before and after learning training. (**A**) Morphology of hippocampal neurons cultured for 1 week in vitro. (**B**) The neurons on the electrodes in (**A**) at higher magnifications. (**C**) The distribution and proportion of electrodes recording neuronal activities in Group 1 (red), electrodes recording neuronal activities in Group 2 (blue), inactive electrodes (gray), and stimulating electrodes (green). (**D**) Spikes and LFPs recorded by the neural sensor before and after training.

After the neurons were cultured in vitro for 1 week, they formed an intricate network through synaptic connections (Figure 5A,B). At this stage, the electrical activity of the neurons could be accurately detected with our sensors. When the neurons were cultured in vitro after 2–3 weeks, stable and synchronized oscillation activities were recorded by the sensors (Figure 5D). Studies have shown that the emergence of synchronized oscillatory activities is a sign of the maturity of physiological functions in high-density neuron cultures [37]. Therefore, learning training experiments were conducted until the neurons exhibited the synchronized oscillation activity. The neurons were cyclically trained through the low-frequency electrical stimulation sequence shown in Figure S2 to construct a learning model. The sensor detected marked changes in the electrical activity of neurons after learning training in the hippocampus culture (Figure 5D).

**Figure 6:**
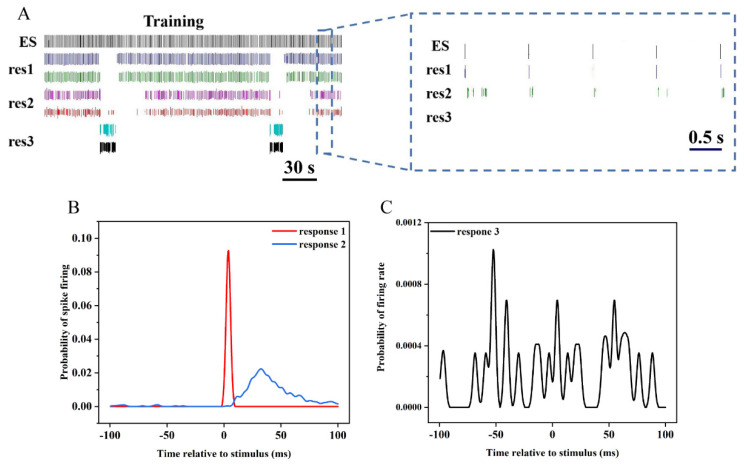
Responses of hippocampal neurons to electrical stimulation. (**A**) Three kinds of representative spike trains of neuronal responses to stimuli during electrical stimulation training. (**B**) The PSTH of two effective responses of neurons to electrical stimulation. (**C**) The PSTH of ineffective responses of neurons to electrical stimulation.

As shown in Figure 6A, we performed a comparison between the time stamps of spike firing and electrical stimulation (ES) to study the neurons’ responses to the electrical stimulation. It was clear that the neural sensors detected three or more distinct responses of neurons to electrical stimulation. Some neurons responded immediately by generating spikes following electrical stimulation after multiple electrical stimulation trainings. The second response was that the discharge of neurons in a short period of time after electrical stimulation increases significantly. The third was that the activity of neurons was not affected by electrical stimulation.

Peri-stimulus time histograms (PSTHs) are a common means used to investigate the correlation between spikes and electrical stimulation [38]. Therefore, we used PSTH to analyze the neural information contained in these three responses (Figure 6B,C). The first kind of firing activity was centered in the first 20 ms after stimulation, which suggested that the sensitivity of ion channels on the cell membrane of the neuron was changed by the stimulation, meaning that the neuron could quickly respond to the stimulation. The second was concentrated in 20–100 ms after the stimulus, which was consistent with the time interval when the synapse responds to the stimulus, indicating that the second response triggered a change in the plasticity of the network. However, the PSTH of the third kind did not change significantly before and after the stimulation, probably due to these neurons’ insensitivity to electrical stimulation.

### 3.5. Effects of Electrical Stimulation on Different Types of Neurons

**Figure 7:**
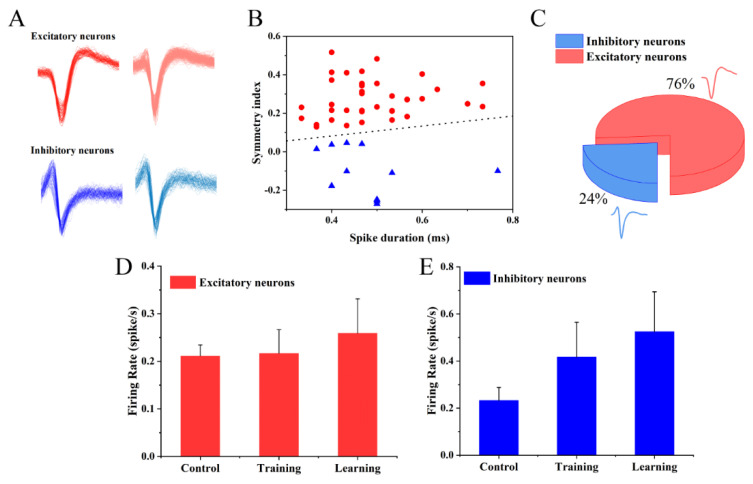
The cellular level changes in electrophysiology of in vitro cultured neurons through learning training. (**A**) Representative spike patterns of detected excitatory and inhibitory neurons. (**B**) The classification of neurons distinguished by the k-means method. (**C**) The ratio of excitatory to inhibitory neurons. (**D**) The responses of excitatory neurons to learning and training. (**E**) The responses of inhibitory neurons to learning and training.

Based on the outstanding electrical properties of the neural sensor, the MEA accurately recorded both excitatory and inhibitory discharge waveforms of neurons (Figure 7A). Therefore, we used k-means cluster analysis to divide the detected 50 spike units into two types based on the differences in the spike duration and symmetry index (Figure 7B and Figure S10) [39]. The results showed that the main difference between type 1 spike units and type 2 is that the symmetry index of type 1 is larger than that of type 2 (Figure 7B). Subsequently, we calculated the ratio of the two spike units (type 1 accounted for 24%, type 2 accounted for 76%) (Figure 7C), which was the same as the reported ratio of excitatory pyramidal neurons and inhibitory interneurons in the hippocampus (inhibitory neurons account for about 20–30%) [40]. Furthermore, we discovered that the two types of waveforms detected were similar to those previously reported for excitatory pyramidal neurons and inhibitory pyramidal neurons in the hippocampus. Thus, we could infer that the type 1 neurons were inhibitory neurons and type 2 neurons were excitatory neurons. As shown in Figure 7D,E, we calculated the effects of learning training on two types of neurons. The results showed that after electrical stimulation, the discharge frequency of both was improved. However, inhibitory neurons were affected more than excitability (the firing rate of the inhibitory neurons increased by 111%, and that of the excitatory neurons increased by only 15.8%). These results also proved that electrical stimulation could change the excitability/inhibition ratio of cultured neurons, thereby influencing the network pathway [41].

### 3.6. Electrophysiological Characteristics at Population Level

**Figure 8:**
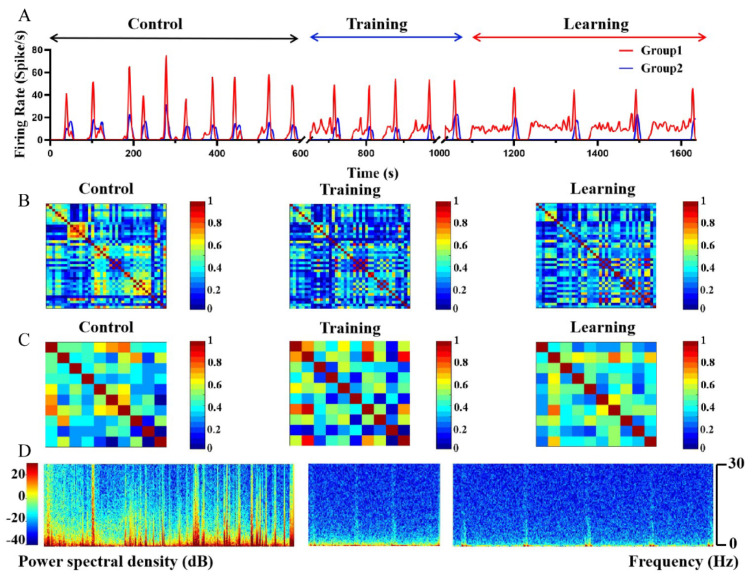
Electrophysiological changes at the network level to neurons cultured in vitro through training. (**A**) The overall firing rate of neurons in the two groups before, during, and after learning. (**B**) The synchrony index among Group 1 neurons before, during, and after training. (**C**) The synchrony index among Group 2 neurons before, during, and after training. (**D**) The power spectrogram of the three stages.

As shown in Figure 5C,D, we divided the microelectrodes on the MEA into inactive and active sites according to whether the microelectrodes recorded neuronal firing. Next, we divided the active sites into two groups according to whether the neurons recorded by sites in the spike panel fired synchronously after electrical stimulation. Subsequently, we marked the recording sites where signals from different groups were detected and analyzed them separately. Through the spike train, the electrophysiological activities of the two groups were found to have a sequential relationship in the time sequence, which was more obvious in the spike train after learning. As shown in Figure 5D and Figure 8A, we could infer that there was neural information transmission between Group 1 and Group 2, and the direction was from Group 1 to Group 2. However, no significant difference was found between the local field potentials (LFP) of the two groups before and after learning, and both showed a trend of changing from chaotic to regular (Figure 5D).

**Figure 9:**
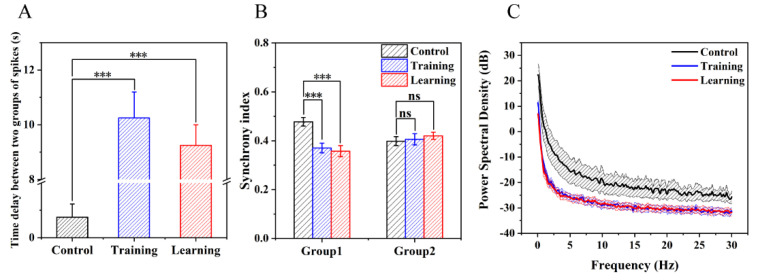
Electrophysiological statistical analysis of two types of neurons at the network level. (**A**) Time delay between spikes of the neurons the two types of neurons. (**B**) The average synchrony index of neurons in excitatory neurons and inhibitory neurons before, during, and after training. (**C**) The power of LFP before and after training (*n* = 5, *** *p* < 0.001).

Subsequently, we analyzed and compared the spikes and LFPs of the neurons in Group 1 and Group 2 in the time and frequency domains before, during, and after training. First, we summed the firing of neurons in the same group before, during, and after training, and finally obtained the firing rates of Group 1 and Group 2 (Figure 8A). Then, we counted and analyzed the time delay of the end time of the bursts in the two groups and found that the delay between the two groups increased from 0.75 ± 0.48 s (control) to 10.25 ± 0.95 s (training) and then recovered to 9.25 ± 0.75 s (learning) (Figure 9A). According to these results, we could theorize that the electrical stimulation training weakened the connection strength of the two groups, which in turn led to the transformation of the information transmission path between the groups.

In the study of neuronal learning induced by electrical stimulation, there is a close relationship between the synchronous firing of neurons and the strength of network connections [42]. As shown in Figure 8B,C, the synchrony coefficients between the neurons of the two networks changed significantly before, during, and after training. Moreover, the change in the synchrony index of the electrical activity of neurons in Group 1 was more obvious than that of Group 2 (Figure 9B). This indicated that the neurons in Group 1 were directly affected by electrical stimulation, while the changes in neurons in Group 2 might have been influenced by the Group 1.

LFP reflects the population activity of the neuron near the recording site; thus, it can also be used to describe network activity [43]. Therefore, LFP was used to study the characteristics of learning at the population level. However, the existing research on neuron learning has rarely focused on this aspect. As shown in Figure 8D, the LFP of Group 1 and Group 2 showed a significant declining trend in both the time domain and the frequency domain during and after training compared to before training. It was found that the power of LFP was reduced from 156.49 ± 22.97 mW (control) to 30.77 ± 5.79 mW (training) (Figure 9C) (*n* = 5, *** *p* < 0.001). The above results showed that the training greatly reduced the non-learning activities of the neural network, and the network more prominently showed the characteristics through training.

## 4. Conclusions

In this research, a neural sensor was fabricated by combining MEMS technology and cell culture techniques. In addition, the monitoring and regulation performance of the three types of neural interfaces modified with Pt, CNT/PEDOT:PSS and cGO/PEDOT:PSS were compared to determine whether they were suitable for in vitro studies of brain learning functions or not. The Pt, CNT/PEDOT:PSS and cGO/PEDOT:PSS C_dl_ were determined by electrochemical methods to be 9.91, 50.24 and 87.11 nF, respectively. This indicates that the cGO/PEDOT:PSS-modified electrode exhibited the largest active area (followed by CNT/PEDOT:PSS and Pt). The increase in active area caused the sensor to have a better neural signal recording ability, which was further manifested by the ultra-low impedance, small phase delay and high response current of the neural interface. In terms of the electrical stimulation performance, we used cyclic voltammetry and voltage transient measurement to evaluate the CSC and CIL of different interfaces. The neural interface based on the cGO/PEDOT:PSS material showed the best performance functions (the charge storage capacity was 7.53 ± 0.34 mC/cm^2^, and the charge injection limit was 3.11 ± 0.25 mC/cm^2^). In addition, the long-term culture of neurons in vitro verified that cGO/PEDOT:PSS modification led to excellent biocompatibility and stability. In the hippocampal neuron learning training experiment, the neural sensor successfully activated the learning function of the hippocampal neurons through electrical stimulation. Moreover, the neural interface with high spatio-temporal resolution not only recorded neural signals at the population level, it also recorded subtle changes at the cell level. As a result, the neural sensor in this study is expected to provide an effective platform for detecting and regulating neurons for studying brain function in vitro.

## Data Availability

Data are contained within the article.

## Supplementary Materials

The following supporting information can be downloaded at: https://www.mdpi.com/article/10.3390/bios12070546/s1, Figure S1: Internal layout and interface circuit of MEA; Figure S2: Electrical stimulation model for the activation of hippocampal neurons to produce learning; Figure S3: SEM images of the three neural interfaces; Figure S4: Measurements of C_dl_ from cyclic voltammetry experiments; Figure S5: The electrical characteristics of the neural sensor; Figure S6: The biocompatibility and stability of cGO/PEDOT:PSS-modified microelectrodes; Figure S7: The safe potential window of Pt and cGO/PEDOT:PSS; Figure S8: Detecting CIL pulsing currents applied to neural interfaces; Figure S9: SEM images of PEDOT:PSS-modified microelectrodes after multiple CV scans; Figure S10: Neuron classification methods; Table S1: Comparison of electrochemical performance of electrodes modified with different materials. References [44,45,46,47,48,49] are cited in the Supplementary Materials.
