# Supplementary material for: A Neural Sensor with a Nanocomposite Interface for the Study of Spike Characteristics of Hippocampal Neurons under Learning Training
Source: Biosensors (Basel). 2022 Jul 21;12(7):546. doi: 10.3390/bios12070546 (PMC9312960; doi:10.3390/bios12070546)
Supplement: Supplementary file 1 [file biosensors-12-00546-s001.zip › biosensors-1804024-supplementary.pdf]

Supplementary Materials

A Neural Sensor with Nanocomposites Interface for the Study of the Spike Characteristics of Hippocampal Neurons under Learning Training

Shihong Xu[1,2], Yu Deng[3], Jinping Luo[1,2], Yaoyao Liu[1,2], Enhui He[1,2], Yan Yang[1,2], Kui Zhang[1,2], Longze Sha[3], Yuchun Dai[1,2], Tao Ming[1,2], Yilin Song[1,2], Luyi Jing[1,2], Qi Xu[3, *], Chengyu Zhuang[4,*], Xinxia Cai[1,2,*]

[a] State Key Laboratory of Transducer Technology, Aerospace Information Research Institute, Chinese Academy of Sciences, Beijing 100190, China.

[b] School of Electronic, Electrical and Communication Engineering, University of Chinese Academy of Sciences, Beijing 100049, China.

[c] State Key Laboratory of Medical Molecular Biology, Institute of Basic Medical Sciences, Chinese Academy of Medical Sciences and Peking Union Medical College, Beijing 100005, China.

[d] Department of Orthopaedics, Ruijin Hospital, Shanghai Jiao Tong University School of Medicine, Shanghai 200025, China.

Figure S1:

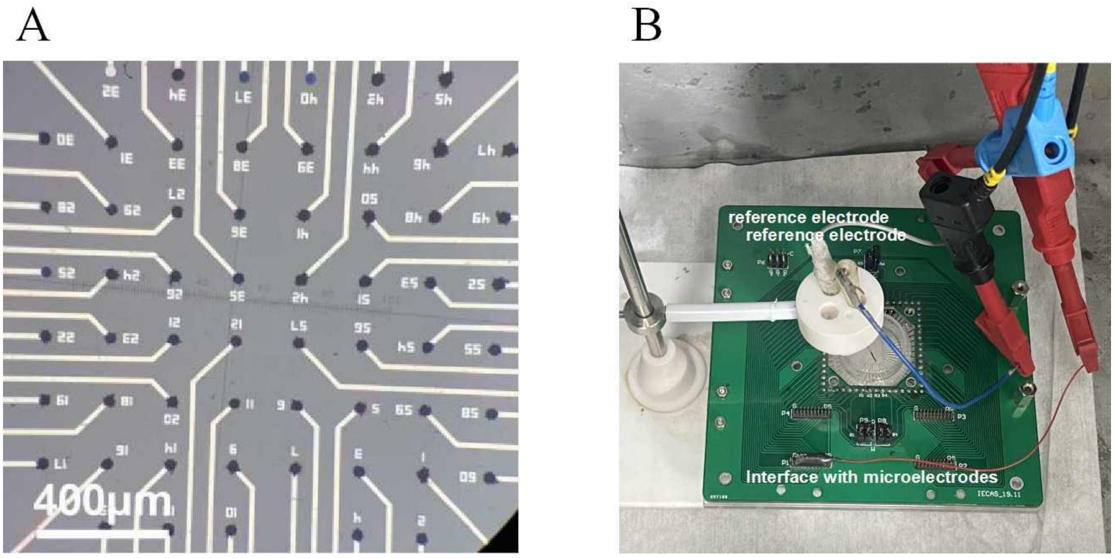

Figure S1. The internal layout and interface circuit of MEA. (A) Internal layout of MEA. (B) The interface circuit of MEA.

Figure S2:

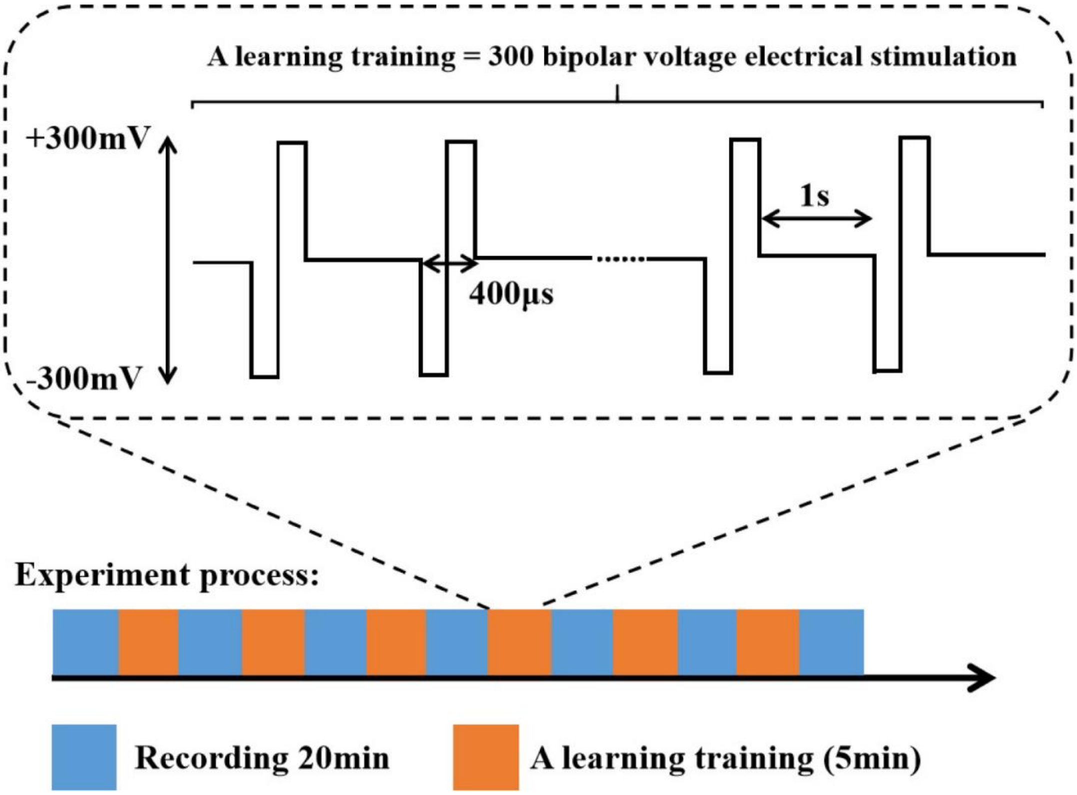

Figure S2. Electrical stimulation model for activation of hippocampal neurons to

produce learning.

Figure S3:

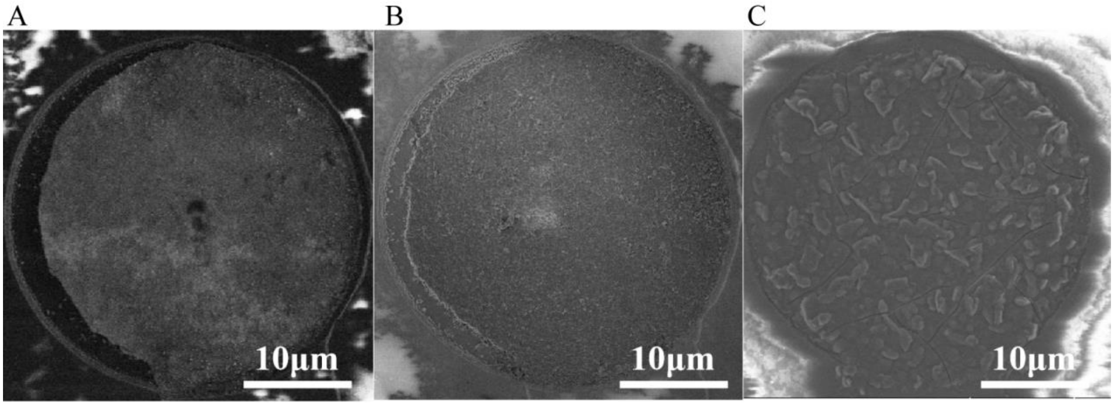

Figure S3. SEM images of three neural interfaces. (A) SEM image of Pt. (B) SEM image of CNT/PEDOT:PSS. (C) SEM image of cGO/PEDOT:PSS.

Figure S4:

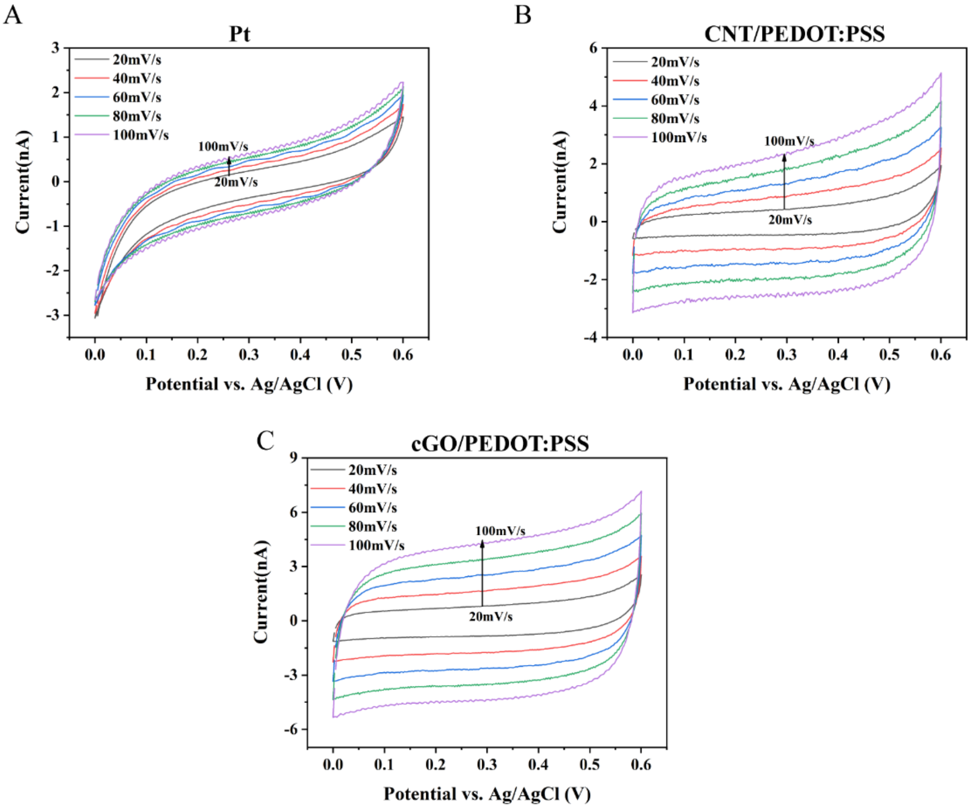

Figure S4. Measurements of Cdl from cyclic voltammetry experiments. (A) The cyclic voltammetry curves of Pt at different scan rates. (B) The cyclic voltammetry curves of CNT/PEDOT:PSS at different scan rates. (C) The cyclic voltammetry curves of cGO/PEDOT:PSS at different scan rates.

Figure S5:

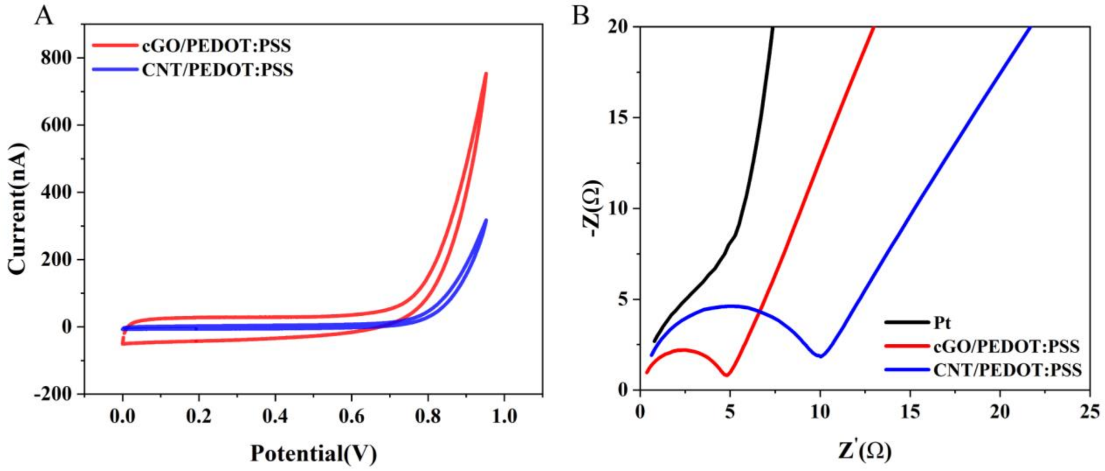

Figure S5. The electrical characteristics of neural sensor. (A) The reaction current of CNT/PEDOT:PSS and cGO/PEDOT:PSS during modification. (B) The Nyquist impedance spectrum of Pt, CNT/PEDOT:PSS and cGO/PEDOT:PSS-modified microelectrodes.

Figure S6:

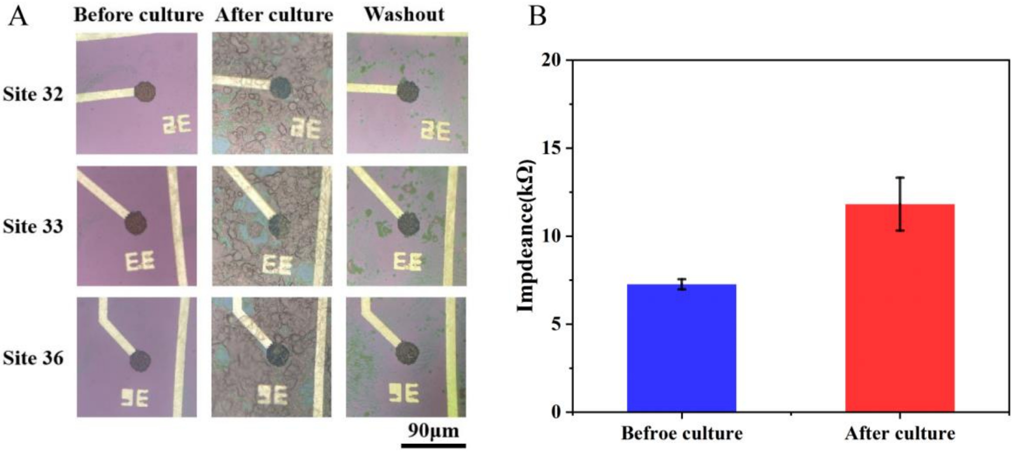

Figure S6. The biocompatibility and stability of cGO/PEDOT:PSS-modified microelectrodes. (A) Changes of coating material (cGO/PEDOT:PSS) morphology before and after cell culture. (B) The impedance of CNT/PEDOT:PSS-modified microelectrodes before and after cell culture (n=5).

Figure S7:

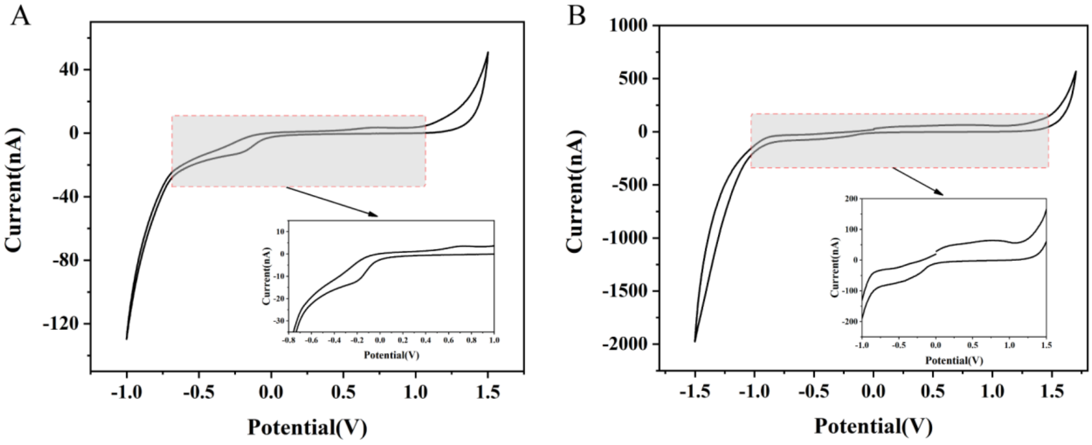

Figure S7. The safe potential window of Pt and cGO/PEDOT:PSS. (A) The safe potential window of Pt. (B) The safe potential window of cGO/PEDOT:PSS

Figure S8:

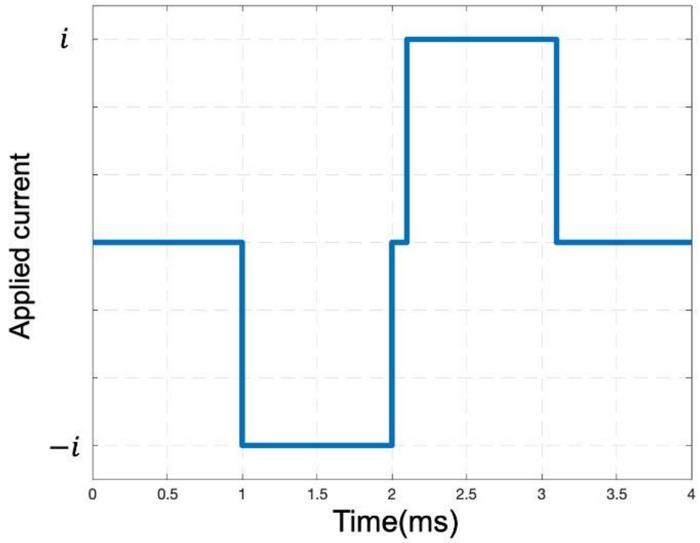

Figure S8. Detecting charge injection limits (CIL) pulsing currents applied to neural interfaces.

Table S1

Table S1. Comparison of electrochemical performance of electrodes modified with different materials.

Electrode material	Geometric area (μ m^2)	|Z| [MΩ μm^2] at 1 kHz	CSC (mC/cm^2)	CIL(mC/cm^2)	Reference
Pt	706.5	393.8	0.37	0.19	This work
Ptlr	4500	452	1.2	0.15	[1]
Au	-	-	0.4	0.4	[2]
TiN	4000	-	2.47	0.9	[3]
Ir	1250	586	0.1	NA	[4]
PtNPs	5000	390	1.2	3	[5]
CNT array	5700	5.9	-	1-1.6	[6]
CNT/PEDOT:PSS	706.5	11.89	4.80	1.93	This work
cGO/PEDOT:PSS	706.5	5.13	7.53	3.11	This work

Figure S9：

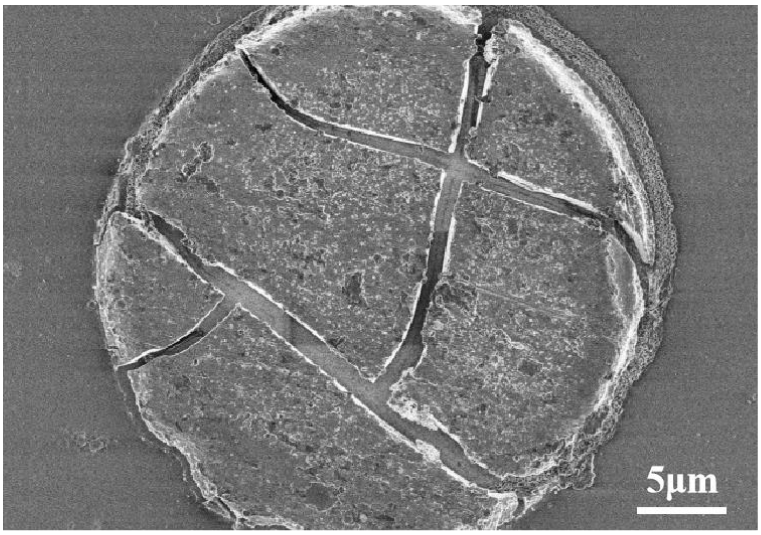

Figure S9. SEM images of PEDOT:PSS-modified microelectrodes after multiple CV scans

Figure S10：

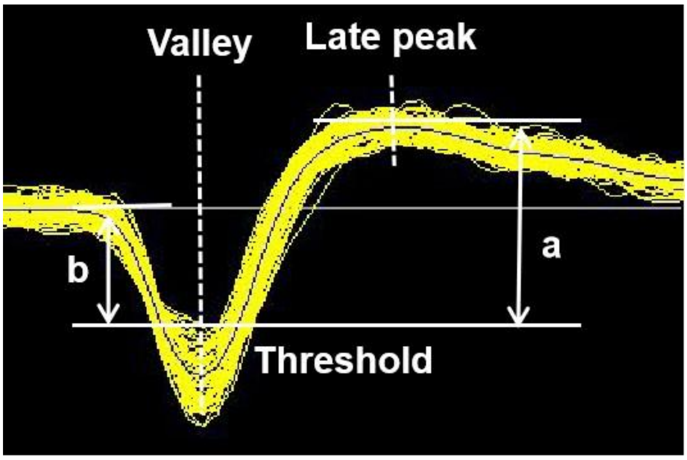

Figure S10. Neuron Classification Methods.

Spike duration = $T_{Late\ peak} - T_{Valley}$
Symmetry index = (a-b)/(a+b)

Reference：

(1) Venkatraman, S.; Hendricks, J.; King, Z. A.; Sereno, A. J.; Richardson-Burns, S.; Martin, D.; Carmena, J. M., In vitro and in vivo evaluation of PEDOT microelectrodes for neural stimulation and recording. IEEE Trans Neural Syst Rehabil Eng 2011, 19 (3), 307-316.

(2) Du, ZJ; Luo, XL; Weaver, CL; Cui, XT. Poly(3,4-ethylenedioxythiophene)-ionic liquid coating improves neural recording and stimulation functionality of MEAs. JOURNAL OF MATERIALS CHEMISTRY C 2015, 3(25), 6515-6524.

(3) Weiland, J. D.; Anderson, D. J.; Humayun, M. S., In vitro electrical properties for iridium oxide versus titanium nitride stimulating electrodes. IEEE Trans Biomed Eng 2002, 49 (12 Pt 2), 1574-1579.

(4) Mohammad Reza Abidian; Joseph M. Corey; Daryl R. Kipke; David C. Martin. Conducting-Polymer Nanotubes Improve Electrical Properties, Mechanical Adhesion, Neural Attachment, and Neurite Outgrowth of Neural Electrodes. Small 2010, 6(3), 421-429.

(5) Wang, K.; Fishman, H. A.; Dai, H. J.; Harris, J. S., Neural stimulation with a carbon nanotube microelectrode array. Nano Letters 2006, 6 (9), 2043-2048.

(6) Park, S.; Song, Y. J.; Boo, H.; Chung, T. D., Nanoporous Pt Microelectrode for Neural Stimulation and Recording: In Vitro Characterization. Journal of Physical Chemistry C 2010, 114 (19), 8721-8726.
